# Successive accumulation of biotic assemblages at a fine spatial scale along glacier-fed waters

**DOI:** 10.1016/j.isci.2024.109476

**Published:** 2024-03-26

**Authors:** Qi Lu, Yongqin Liu, Jindong Zhao, Meng Yao

**Affiliations:** 1 School of Life Sciences, Peking University, Beijing 100871, China; 2 Institute of Ecology, College of Urban and Environmental Sciences, Peking University, Beijing 100871, China; 3 Center for Pan-Third Pole Environment, Lanzhou University, Lanzhou 730000, China; 4 State Key Laboratory of Tibetan Plateau Earth System, Resources and Environment (TPESRE), Institute of Tibetan Plateau Research, Chinese Academy of Sciences, Beijing 100101, China; 5 University of Chinese Academy of Sciences, Beijing 101408, China

**Keywords:** Environmental science, Ecology, Environmental Biotechnology

## Abstract

Glacier-fed waters create strong environmental filtering for biota, whereby different organisms may assume distinct distribution patterns. By using environmental DNA-based metabarcoding, we investigated the multi-group biodiversity distribution patterns of the Parlung No. 4 Glacier, on the Tibetan Plateau. Altogether, 642 taxa were identified from the meltwater stream and the downstream Ranwu Lake, including 125 cyanobacteria, 316 diatom, 183 invertebrate, and 18 vertebrate taxa. As the distance increased from the glacier terminus, community complexity increased via sequential occurrences of cyanobacteria, diatoms, invertebrates, and vertebrates, as well as increasing taxa numbers. The stream and lake showed different community compositions and distinct taxa. Furthermore, the correlations with environmental factors and community assembly mechanisms showed group- and habitat-specific patterns. Our results reveal the rapid spatial succession and increasing community complexity along glacial flowpaths and highlight the varying adaptivity of different organisms, while also providing insight into the ecosystem responses to global change.

## Highlights

• eDNA captured multitrophic biodiversity along glacial waters
• Biological communities showed spatial succession within a short distance
• Cyanobacteria, diatoms, invertebrates, and vertebrates occurred sequentially
• Different factors drive the assembly of different biological groups

## Graphical abstract

**Figure:**
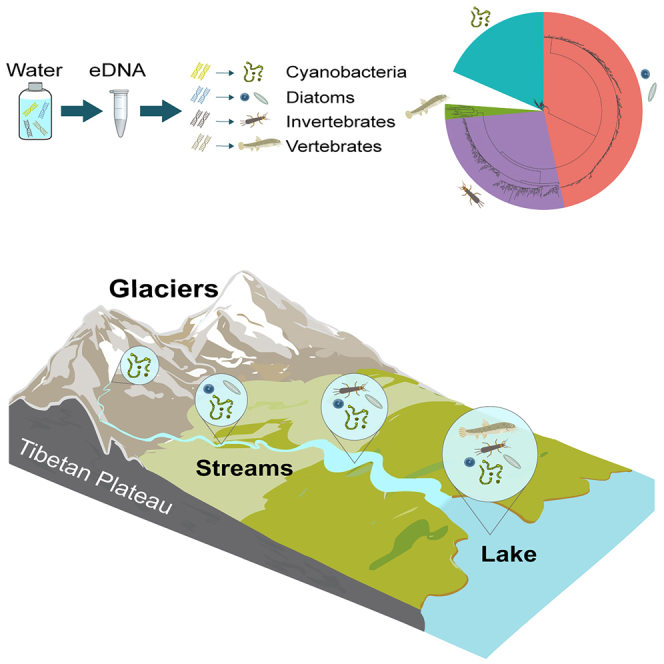

## Introduction

Glaciers hold the main freshwater resources on Earth and play pivotal roles in regulating regional hydrology and climate.^1^^,^^2^ Additionally, the world’s glaciers are among the most sensitive systems to climate change, and many are receding in high-latitude and high-altitude zones,^3^^,^^4^^,^^5^ a trend that is seriously threatening the associated biodiversity, ecological functions, and ecosystem services for hundreds of millions of people.^6^^,^^7^^,^^8^ Therefore, there is an urgent need to understand the ecological processes of these systems to predict the impacts of glacial retreat on diverse biota and ecosystem dynamics.

Glacier-fed waters are extreme, dynamic, and heterogeneous systems. From an ecological perspective, the low temperature, low atmospheric oxygen, high ultraviolet radiation, frequent disturbance, and ultraoligotrophic status of glacier-fed aquatic systems stress organisms in many ways and create strong environmental filtering for the taxonomic lineages and biological traits of those living in such habitats.^6^^,^^9^ Those environmental stressors are particularly prominent near the glacier terminus and result in biodiversity “coldspots” that have low species numbers and productivity.^8^^,^^10^ As glacial influences weaken with distance along the flowpath, glacier-fed waters progress through diverse hydrological and physiochemical features that allow them to ultimately support a highly diverse community of organisms.^6^^,^^11^ Indeed, different biota form spatially distinct communities in and around glacial waterways, thus demonstrating how the variable inhabitability of glacier-influenced environments correlates with the distinct adaptivity of specific taxa.^12^ While the ways different biological groups assemble and organize along glacier-fed waters are not well understood, the sensitivity and tolerance of organisms to abiotic environments and their distinct requirements for biotic conditions are known to vary.^13^^,^^14^ Resolving the biodiversity and community compositions along glacial waterways will provide vital information about the processes and drivers of community structures in glacier-influenced aquatic ecosystems. Such knowledge can also aid understanding of biological community organization in other extreme environments^15^ and help elucidate taxa-specific responses and ecosystem alterations under climate change.^6^

The traditional methods of performing comprehensive biodiversity assessments are daunting tasks fraught with logistical obstacles because of the need for diverse survey methods and extensively varied expertise in morphotaxonomic identification across vastly different biological groups. The already strenuous task of traditional biodiversity surveys is made even more difficult when coupled with the remote and harsh environmental conditions of most glaciers. Therefore, most studies of glacier-influenced biota have addressed a single biological group (e.g., bacteria, arthropods, and fish). However, recent technological advances in environmental DNA (eDNA) methodology have allowed non-invasive, robust, and economic surveys of diverse organisms via the detection of trace amounts of their DNA in environmental samples (e.g., water, soil, and ice).^16^ This technology, further integrated with high-throughput sequencing of amplicons of the DNA fragments that allow taxon identification (i.e., DNA barcodes), has yielded the eDNA metabarcoding approach, which has opened an innovative research avenue for comprehensive and standardized biodiversity surveillance at the ecosystem-level in challenging environments.^17^^,^^18^

**Figure 1:**
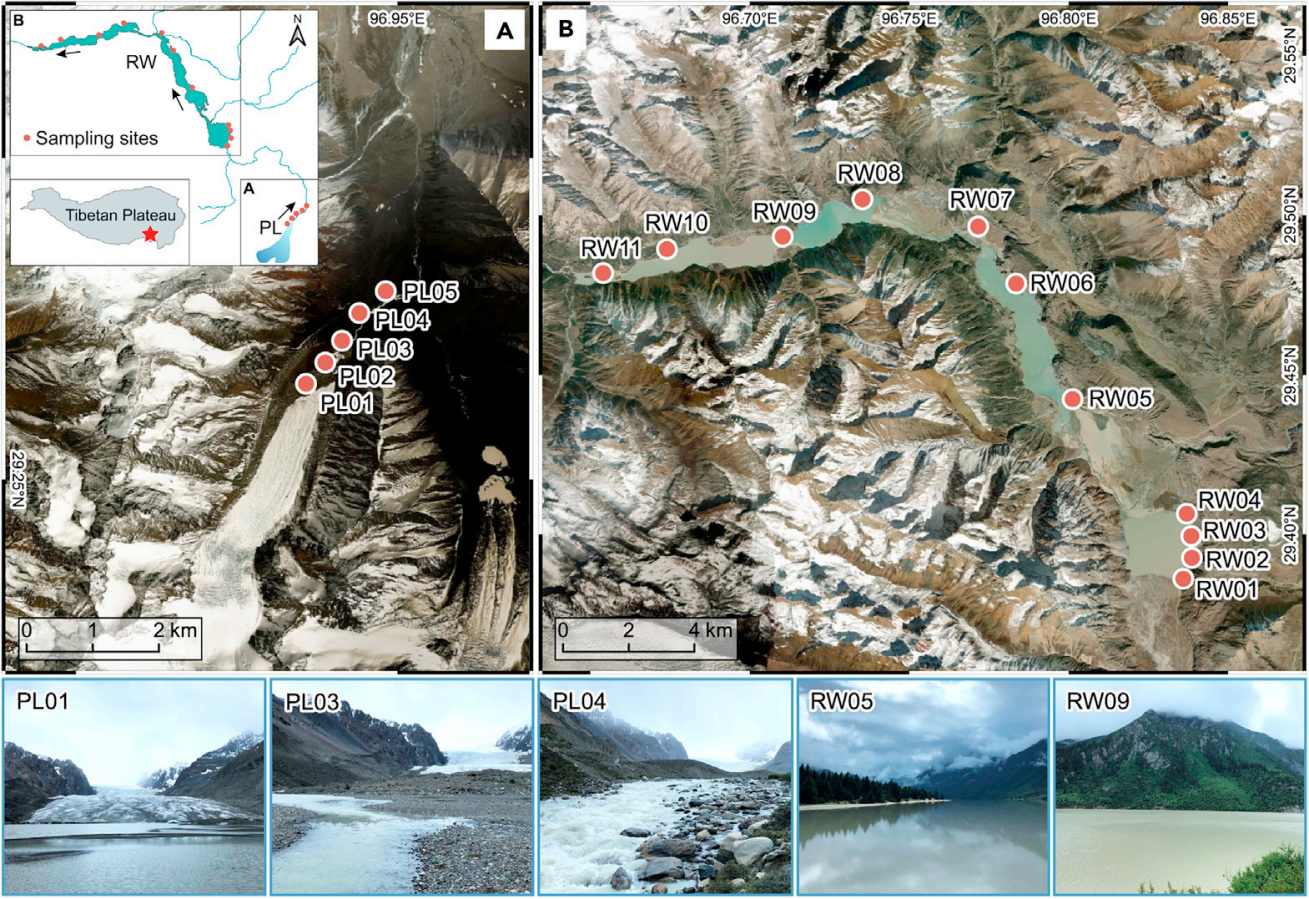
Maps and photographs of the Parlung No. 4 glacier study area and sampling sites (red circles) on the Tibetan Plateau (A) Proglacial sites (PL01–PL05) at the glacier’s terminus and along the glacier-fed stream. (B) Lake sites (RW01–RW11) along the glacier’s downstream lake, Ranwu. The geological maps were generated using QGIS v.3.22 (https://qgis.org) with an ESRI World Imagery base layer. The insets for (A) and (B) show the geographic placement of the glacier and lake, and the red star indicates the study area. Arrows denote the direction of flow. PL, Parlung No. 4 glacier; RW, Ranwu Lake. Photos show the environments of representative sampling sites.

The Tibetan Plateau holds the world’s largest volume of glaciers outside the poles.^19^ However, it is also one of the most sensitive regions to climate warming and its glaciers are melting at accelerating rates.^20^^,^^21^^,^^22^^,^^23^ Yet, we still know little about the biological community structures and ecological processes of most glacier-fed waters in this region. Located on the Tibetan Plateau, the Parlung No. 4 glacier (PL; Figure 1) is one of the sources of the Parlung-Zangbo River, the largest tributary of the Brahmaputra River. It is a regional benchmark glacier because its meteorology and mass balance have been well-monitored, and it, as with many other glaciers on the Tibetan Plateau, has been rapidly losing mass over the past 15 years.^24^ The PL-fed systems are characterized by high-altitude, barren lands with no human activity at the glacier’s terminus, but with gradually increasing riparian vegetation, human activity, and livestock grazing along the meltwater streams and around the downstream lake, Ranwu (Figure 1; Table S1). Here, to understand how biological communities organize in response to heterogeneous glacial waterway conditions, we used a multi-marker eDNA metabarcoding approach to analyze the biodiversity distributions of major biological groups. Four broad groups were analyzed, each representing distinct phylogenetic, morphological, and trophic characteristics: cyanobacteria (prokaryotic producers), diatoms (eukaryotic producers), invertebrates (small-bodied lower-level consumers), and vertebrates (large-bodied higher-level consumers). Collectively, these four groups comprise the majority of life forms in the selected ecosystem. Multi-group community compositions were examined at a fine spatial scale along the glacier-fed waters, ranging from the proglacial lake at PL’s terminus, through the adjacent meltwater stream, and around Ranwu Lake. The aims of our study were to (1) unveil the fine-scaled distribution patterns and assemblage compositions of different biota in PL-fed aquatic ecosystems, (2) investigate the structural variation of biological communities along PL’s glacial flowpath, and (3) assess the effects of environmental factors and various community assembly processes on different biota in PL-influenced waters.

## Results

### DNA sequencing and taxonomic assignments

We used four metabarcoding primer sets to amplify eDNA extracts, with each targeting a specific biological group’s gene regions: the cyanobacterial 16S rRNA gene (CYA),^25^ the diatom rbcL gene (708F),^26^ the invertebrate COI gene (BF),^27^ and the vertebrate 12S rRNA gene (Tele02)^28^ (Table S2). We obtained 127,474,482 raw reads from all sequencing libraries, of which 6,955,873 (cyanobacteria 737,282; diatoms 4,482,675; invertebrates 35,206; vertebrates 1,700,710) remained after stringent quality filtering and bioinformatics processing. The sequences were clustered into 642 operational taxonomic units (OTUs); 125 OTUs were assigned to cyanobacteria, 316 OTUs to diatoms, 183 OTUs to invertebrates, and 18 OTUs to vertebrates (Table S3). The rarefaction curves demonstrated that the OTU accumulations in most of the samples reached an asymptote (Figure S1), indicating that those OTUs sufficiently represented their respective assemblages. Subsequently, the resulting OTU reads were normalized to generate quantitative (i.e., relative read abundance [RRA]) data for further analyses (Data S1).

Among the 125 cyanobacterial OTUs, 90 (72.0% OTUs, 82.1% RRA) were classified at the family or lower taxonomic level (Table S3), and 16 families within 12 orders were identified (Table S4). Of the 316 diatom OTUs, 245 (77.5% OTUs, 88.2% RRA) were successfully classified to the family or lower taxonomic level, and those taxonomic assignments revealed 20 families belonging to 10 orders. Of the 183 invertebrate OTUs, 135 (73.7% OTUs, 63.4% RRA) were classified at the order or lower taxonomic level, and a total of 21 orders within 14 classes were identified. For vertebrates, all 18 OTUs were successfully assigned to the family or lower taxonomic level, and 11 families within eight orders were identified.

### Multi-group community compositions at proglacial and lake sites

**Figure 2:**
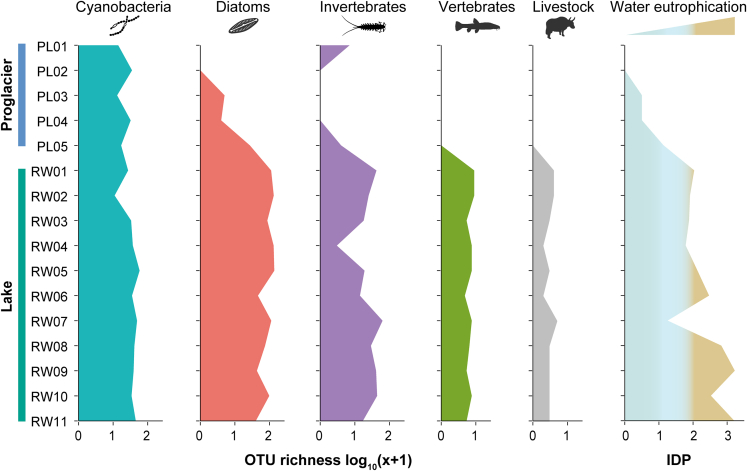
Operational taxonomic unit (OTU) richness (i.e., number of OTUs) distributions of the four biological groups detected at the proglacial (PL01–PL05) and lake (RW01–RW11) sites Vertebrate OTUs included all detected vertebrates, and the richness of domesticated species (cattle, yak, horse, sheep, and chicken) was plotted separately from wild taxa as an indicator of human activity. The scale is log-transformed to facilitate visualization of lower values. The Pampean Diatom Index (IDP) reflects the level of organic pollution and eutrophication. For IDP, light green, light blue, and brown indicate low, moderate, and high nutrient levels, respectively.

**Figure 3:**
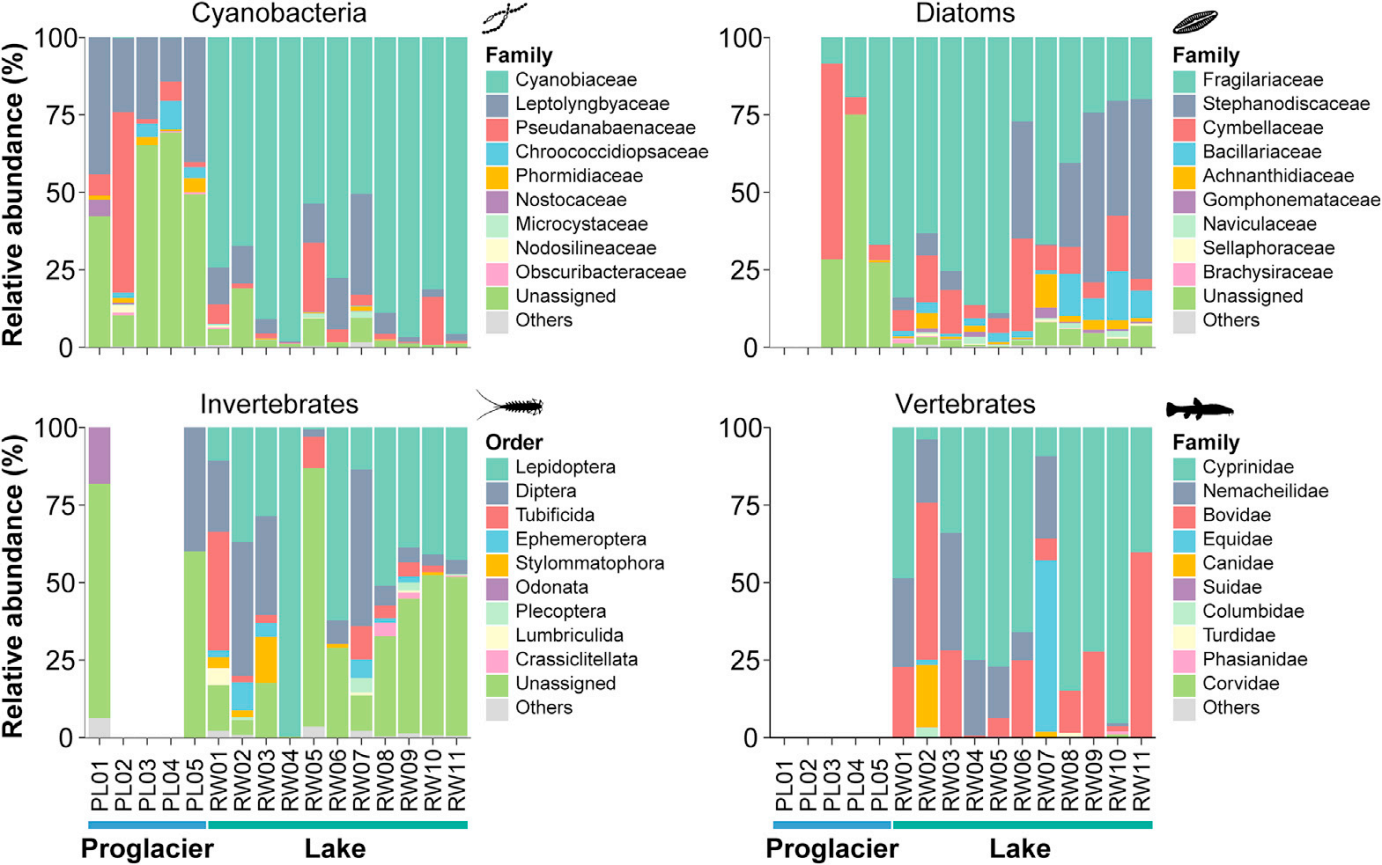
Relative sequence abundance of identified operational taxonomic units (OTUs) for four biological groups along the proglacial headwaters and lake continuum The taxonomic levels displayed are based on more than 70% of the OTUs identified for each group having taxonomic information at that level. The figures depict the 10 most abundant families of cyanobacteria, diatoms, and vertebrates, and the 10 most abundant orders of invertebrates. Low abundance families/orders were collapsed into “Others.” The OTUs that could not be assigned to a single family (for cyanobacteria and diatoms) or order (for invertebrates) were grouped and are shown as “Unassigned.” PCR results for certain proglacial sites were excluded due to low read abundance after sequence filtering, thus explaining the blank sections on the plots.

Cyanobacteria were detected at all sampling sites, with an average 30.5 (range 10–57) OTUs recovered per site (Figure 2; Table S5). At the family level, the cyanobacterial assemblages were dominated by Cyanobiaceae (mean RRA: 54.7%), followed by Leptolyngbyaceae (15.8%) and Pseudanabaenaceae (8.3%), across the sampling sites (Figure 3). Notably, 17.9% of all detected cyanobacterial sequences could not be assigned to a specific cyanobacterial family and were categorized as “Unassigned.” There were noticeable differences in the relative contributions of various cyanobacterial families between the proglacial and lake samples, with Leptolyngbyaceae and Pseudanabaenaceae predominating in the proglacial samples, whereas Cyanobiaceae was most prevalent among family-assigned OTUs in the lake samples (Figure 3).

Diatoms were recovered at all lake sites and in the glacier-fed stream (PL03–PL05), but not at the first two proglacial sites near the glacier terminus (PL01 and PL02). While the taxonomic richness of diatoms was also low in the glacial stream (4, 3, and 26 OTUs detected at PL03, PL04, and PL05, respectively), it was greater in each lake sample (OTU range 42–136, mean = 95.9) (Figure 2). Across all sites, the diatom assemblages were dominated by Fragilariaceae (mean RRA: 49.4%), followed by Stephanodiscaceae (16.3%) and Cymbellaceae (13.7%). Proglacial and lake samples again showed different compositions of diatom taxa, and OTUs unassigned at the family level yet again accounted for large proportions of the proglacial assemblages (Figure 3). Among the family-assigned OTUs, Cymbellaceae dominated at the first proglacial site where diatoms were detected (PL03), while the proportion of Fragilariaceae gradually increased toward the downstream sites. In contrast, Fragilariaceae was the overall most abundant family across the lake samples, but Stephanodiscaceae increasingly dominated the lower lake sites close to the outflow.

Invertebrates were detected in all lake samples, but they were detected only at the first and last proglacial sites (PL01 and PL05). As with diatoms, the invertebrate taxonomic richness was low at the proglacial sites (6 and 3 OTUs found at PL01 and PL05, respectively), but it was generally higher in the lake samples (OTU range 2–62, mean = 27.2) (Figure 2). The assigned invertebrate taxa at the order level were dominated by Lepidoptera (mean RRA: 32.8%), Diptera (16.8%), and Tubificida (5.7%) across all sites (Figure 3). The only OTUs that were assigned to the order or lower levels at the proglacial sites were Odonata at PL01 and Diptera (family Chironomidae) at PL05. However, OTUs unassigned at the order level accounted for an average 36.6% of invertebrate sequences across sites and were prevalent in both proglacial and lake assemblages.

No vertebrate sequences were recovered from any of the proglacial samples, but they were found at all lake sites (3–8 OTUs per site; Figure 2). The dominant taxa within the vertebrate assemblages were fish (6 OTUs), specifically the carp family Cyprinidae (subfamily Schizothoracinae in particular; mean RRA = 55.1%) and the stone loach family Nemacheilidae (genus *Triplophysa*; 15.0%), which are prevalent in the region’s fish assemblages. The six mammalian OTUs consisted mostly of livestock (cattle *Bos taurus*, yak *B. grunniens*, horse *Equus caballus*, and sheep *Ovis aries*), and the six detected avian OTUs included both locally occurring wild birds (blood pheasant *Ithaginis cruentus*, red-billed chough *Pyrrhocorax pyrrhocorax*, white-breasted waterhen *Amaurornis phoenicurus*, pigeon *Columbia* spp., and thrush *Turdus* spp.) and a domesticated species, the chicken *Gallus gallus* (Data S1). These findings demonstrate the ability of eDNA analysis to capture both aquatic and terrestrial species’ signals in water samples that may be accumulated via direct deposit and surface runoff from surrounding areas. Domesticated species accounted for large proportions of vertebrate OTUs at all lake sites (Figure 2), revealing the considerable influence of human activities around the lake.

Pearson correlation analyses revealed that the α diversity (i.e., the number of OTUs detected at each site; Table S5) of all biological groups increased significantly as the collection sites progressed from the proglacial headwater to the lower lake (Pearson: *r* = 0.64, *p* = 0.008 for cyanobacteria; *r* = 0.63, *p* = 0.036 for diatoms; *r* = 0.70, *p* = 0.026 for invertebrates; *r* = 0.58, *p* = 0.019 for vertebrates).

The degree of water eutrophication, as indicated by the diatom-based indicator Pampean Diatom Index (IDP) (see method details), also exhibited a similar increasing trend with the direction of flow (Pearson *r* = 0.90, *p* < 0.001). The low IDPs (0.5–1.1) of the proglacial sites where diatoms were detected indicated good water quality with low nutrient levels, but the higher IDP values (i.e., >2) of the lake samples, particularly the lower lake sites (RW08–RW11) (Figure 2), indicated poorer water quality with higher nutrient levels.^29^ The higher IDP values at the lake sites compared to the proglacial sites were also correlated with strong eDNA signals of domesticated animals in the lake samples. Only one lake site (RW07), located near the inflow of another glacial stream (Figure 1), had a low IDP.

### Differences between proglacial and lake communities

**Figure 4:**
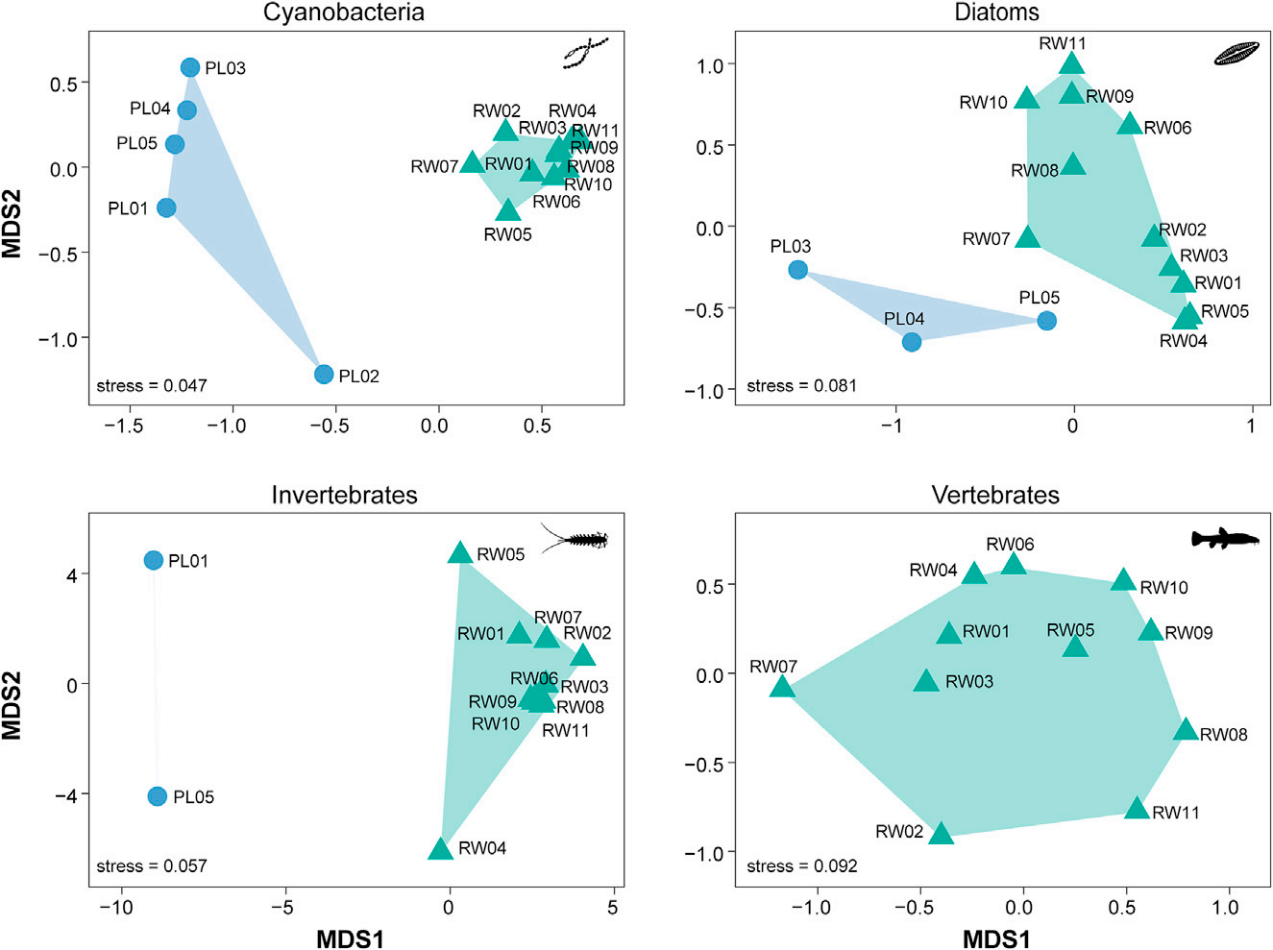
Bray-Curtis dissimilarity-based non-metric multidimensional scaling (NMDS) plots of the four biological groups detected in different habitats Blue circles indicate samples from proglacial sites. Green triangles indicate samples from lake sites.

The non-metric multidimensional scaling (NMDS) analysis of the cyanobacteria, diatoms, and invertebrates revealed a consistent clustering pattern wherein the assemblages from the proglacial and lake sites were clearly separated (Figure 4). Furthermore, the cyanobacteria (permutational multivariate analysis of variance [PERMANOVA]: pseudo-*F*_1,14_ = 26.5, *R*^*2*^ = 0.65, *p* < 0.001), diatom (pseudo-*F*_1,12_ = 4.48, *R*^*2*^ = 0.27, *p* = 0.025), and invertebrate (pseudo-*F*_1,11_ = 2.22, *R*^*2*^ = 0.17, *p* = 0.012) assemblage compositions differed significantly between the proglacial and lake samples.

**Figure 5:**
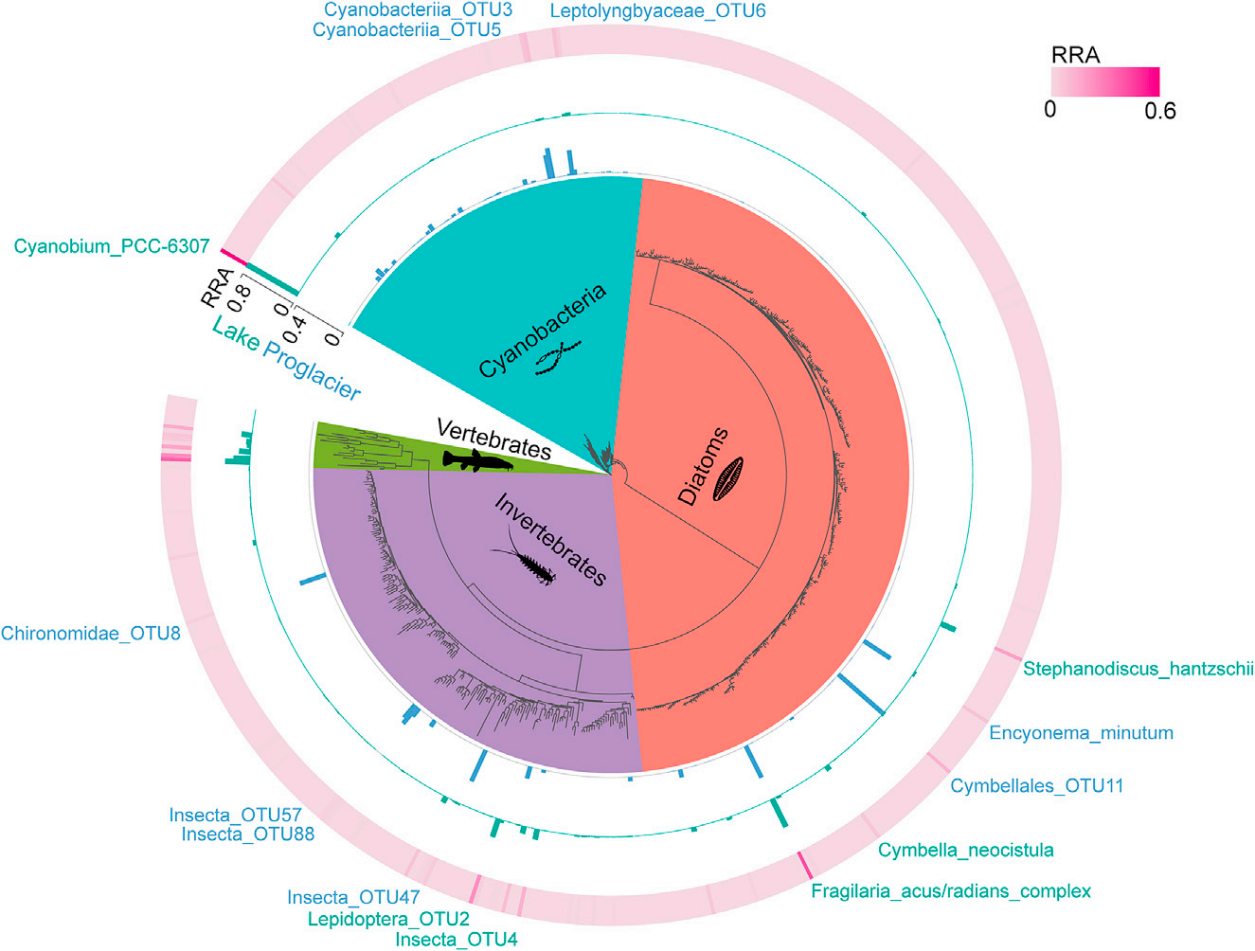
Phylogenetic trees in the pie chart are based on 642 OTUs detected for four biological groups The inner bar graphs represent the OTU relative read abundance (RRA) in the proglacial (blue) and lake (green) samples. The outer ring indicates the mean OTU RRA across all sites, and the names of the OTUs that contributed ≥5% to differences between the glacier and lake assemblages are shown in the color corresponding to their more prevalent habitat.

We more deeply investigated the differential representation of individual OTUs between the proglacial and lake assemblages to identify species that showed strong habitat specificity and were thus main drivers of community turnover. For cyanobacteria, Cyanobacteriia_OTU3, Leptolyngbyaceae_OTU6, and Cyanobacteriia_OTU5 were more abundant in proglacial samples in comparison with lake samples, while *Cyanobium*_PCC-6307 showed the opposite trend (Figure 5). Further analysis based on the similarity percentage (SIMPER) showed that *Cyanobium*_PCC-6307 accounted for most of the dissimilarity between the proglacial and lake assemblages (contribution = 43.8%; Table S6).

For diatoms, Cymbellales_OTU11 and *Encyonema minutum* were more abundant in the proglacial samples, whereas the *Fragilaria acus*/*radians* complex, *Stephanodiscus hantzschii*, and *Cymbella neocistula* were more abundant in the lake samples (Figure 5). SIMPER results showed that Cymbellales_OTU11 and the *Fragilaria acus*/*radians* complex accounted for most of the dissimilarity between proglacial and lake assemblages (Table S6).

For invertebrates, Insecta_OTU47, Chironomidae_OTU8, Insecta_OTU88, and Insecta_OTU57 were the top four most abundant OTUs in proglacial samples. Lepidoptera_OTU2 and Insecta_OTU4 were found only in lake samples and were the two most abundant lake taxa (Figure 5). Lepidoptera_OTU2, Insecta_OTU47, and Chironomidae_OTU8 were the top-ranked OTUs responsible for dissimilarity between the proglacial and lake assemblages.

### Community spatial variations and associated environmental factors

For all biological groups, turnover components dominated the overall β-diversity (Sørensen dissimilarity = 0.744–0.923; Bray-Curtis dissimilarity = 0.813–0.931) among sites, with 75.9%–92.4% of the Sørensen (qualitative) dissimilarity and more than 99.0% of the Bray-Curtis (quantitative) dissimilarity belonging to turnover components (Figure S2).

**Figure 6:**
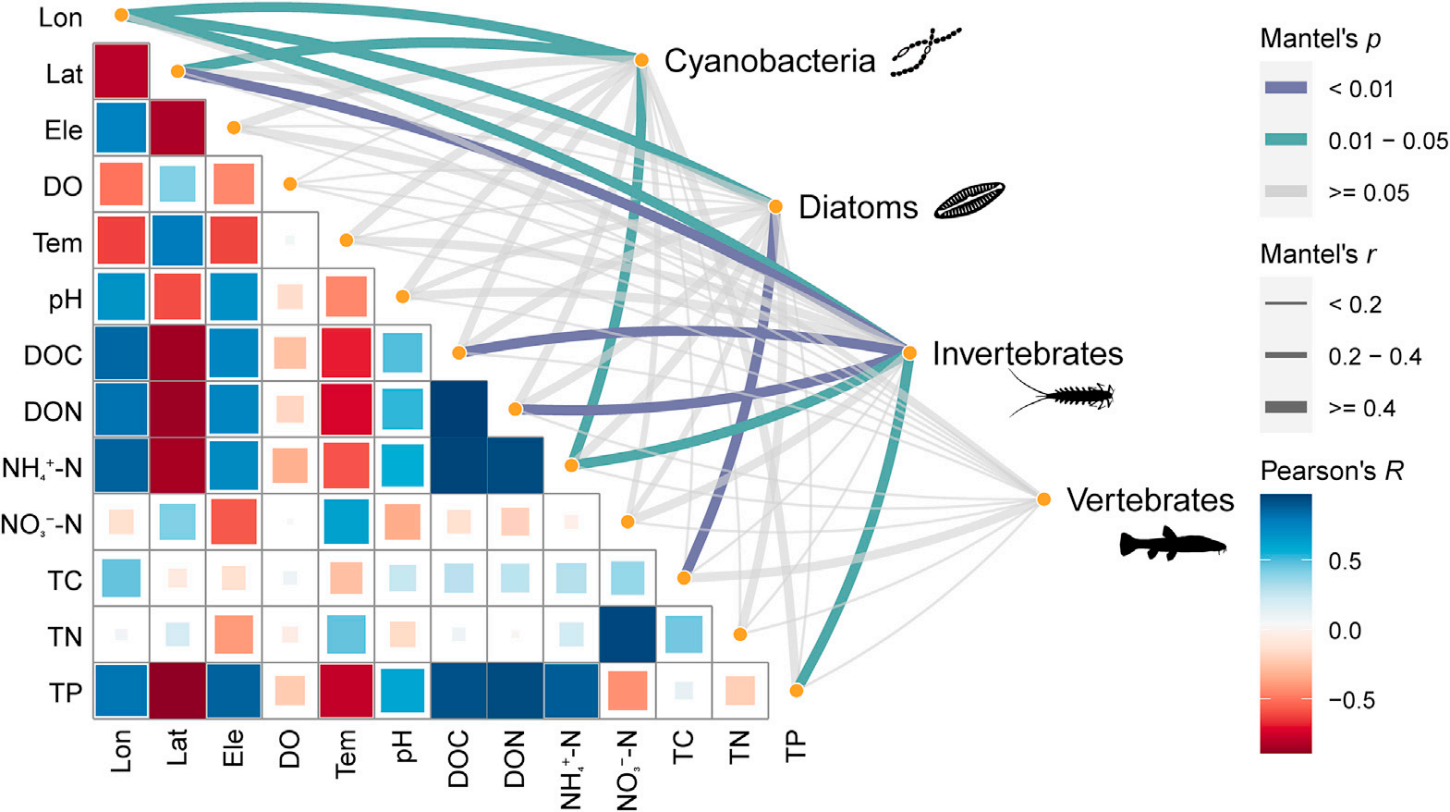
Heatmap of correlations (Mantel tests) between the four biological groups’ assemblage compositions and individual environmental factors The width and color of the edges represent the Mantel’s *r* value and statistical significance, respectively. Pairwise Pearson correlations between environmental factors are indicated with a color gradient. Lon, longitude; Lat, latitude; Ele, elevation; DO, dissolved oxygen; Tem, water temperature; DOC, dissolved organic carbon; DON, dissolved organic nitrogen; NH_4_^+^-N, ammonium nitrogen; NO_3_^−^-N, nitrate nitrogen; TC, total carbon; TN, total nitrogen; TP, total phosphorus.

The compositions of different biological groups showed distinct correlational patterns with environmental variables. Longitude, latitude, and ammonium nitrogen (NH_4_^+^-N) were the main environmental factors correlated with cyanobacterial assemblage composition (Mantel’s *r* = 0.58–0.94, all *p* < 0.05). Longitude (*r* = 0.72, *p* = 0.025) and total carbon (*r* = 0.58, *p* = 0.008) were significantly correlated with diatom assemblages. Invertebrate assemblages were significantly associated with six of the 12 environmental factors, including longitude, latitude, dissolved organic carbon, dissolved organic nitrogen, NH_4_^+^-N, and total phosphorus (*r* = 0.68–0.95, all *p* < 0.05). None of the evaluated environmental factors had significant relationships with vertebrate assemblages (Figure 6).

### Ecological assembly processes of different groups

**Figure 7:**
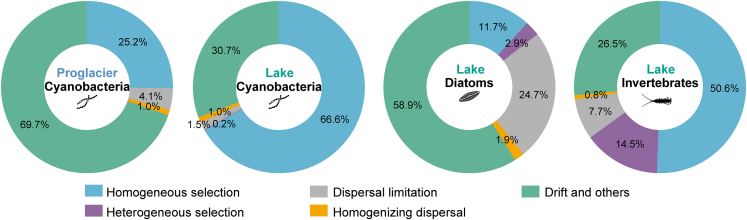
Relative importance of different community assembly processes for cyanobacteria, diatoms, and invertebrates in proglacial and lake habitats The analysis focused solely on cyanobacteria in the proglacial habitat because the sample availability for the other groups was limited.

The community assembly mechanisms of different biological groups were assessed using iCAMP, which revealed that the relative contributions of the community assembly processes (selection, dispersal, and drift) varied among different habitats and groups (Figure 7). For cyanobacteria, community assembly at proglacial sites was predominantly influenced by drift (69.7%), followed by homogeneous selection (25.2%), whereas homogeneous selection (66.6%) had a larger impact than drift (30.7%) at lake sites. For diatoms at lake sites, the community assembly was driven primarily by drift (58.9%), followed by dispersal limitation (24.7%). The community assembly of invertebrates at lake sites was influenced primarily by homogeneous selection (50.6%), followed by drift (26.5%) and heterogeneous selection (14.5%).

## Discussion

### Biodiversity composition and community build-up in glacier-fed waters

This study is the first, to our knowledge, to report the sequential accumulation of various biological groups as the distance from a glacier increases, together with a gradual increase in community composition complexity along glacial meltwater flowpaths, within a small geographic range (∼1.8 km from PL01 to PL05). Our findings highlight the spatial heterogeneity of biodiversity in glacier-fed waters, while illuminating the fine-scaled ecological succession of biological communities under varying levels of glacial influence. Furthermore, our results reveal the differences in environmental tolerance and adaptability among biological groups and taxa.

Cyanobacteria were the only group detected at all sites across the sampled proglacial waters. The pervasiveness of cyanobacteria was expected, because they are known to constitute an important component of microbial communities in cryosphere habitats, such as glaciers and Arctic soils.^30^^,^^31^ Cyanobacteria are often the main primary producers and contributors to both carbon and nitrogen fixation in extreme environments, where they act as ecosystem engineers that also provide critical organic matter for other heterotrophic microbes.^31^^,^^32^^,^^33^^,^^34^ We found that the Leptolyngbyaceae and Pseudanabaenaceae families were most prevalent in proglacial samples, which contrasted with the Cyanobaceae-dominated lake assemblages (Figure 3). The remarkable turnover in assemblage composition suggests that divergent environmental filtering effects are exerted on cyanobacteria in different glacier-influenced habitats, while revealing the ecological adaptivity of each habitat’s characteristic taxa. Leptolyngbyaceae was the most prevalent cyanobacteria in supraglacial sediments/soil from the Arctic archipelago of Svalbard^35^^,^^36^ and in glacier cryoconite from the Tien Shan Mountains in China.^37^ Pseudanabaenaceae was one of the most common cyanobacteria families in cryoconite from polar and mountain glaciers around the globe^38^ and in soils from glacial forefields in both Alaska and Peru.^39^ Our finding that Leptolyngbyaceae and Pseudanabaenaceae were abundant in glacial meltwaters on the Tibetan Plateau suggests universally convergent selection of cyanobacteria lineages in glacial ecosystems. Because glacier meltwater may harbor autochthonous bacteria, as well as allochthonous microbial communities that originate from the surrounding snow, ice, rocks, and soil,^40^ further investigations of microbial community dynamics could shed light on potential ecological linkages between different glacial habitat compartments.

Diatoms, the second most omnipresent group in this study, were found in all sampled waters except for two proglacial sites near PL’s terminus. Few studies have investigated diatoms in meltwaters at the glacier terminus. While diverse diatom taxa have been found in glacier cryoconite holes, their low abundance in that habitat is suggestive of allochthonous deposition rather than habitation by biological communities.^41^^,^^42^ Diatom taxa diversity gradually increased in the meltwater stream with distance from the glacier and reached high richness downstream in Ranwu Lake, suggesting that the harsh physiochemical properties and ultraoligotrophic conditions at PL’s terminus may inhibit diatoms’ stable inhabitance. Interestingly, several dominant diatom taxa in our stream samples (i.e., *Encyonema minutum*, *Hannaea arcus*, and the genera *Cymbella* and *Fragilaria*; Figure 5) were also prevalent in Canadian glacier-fed streams,^43^^,^^44^ suggesting that glacial diatoms undergo conserved environmental filtering around the globe. Diatoms often dominate freshwater algal communities, and they are the main eukaryotic primary producers in alpine fluvial ecosystems.^45^^,^^46^ Hence, high levels of diatom richness and abundance may be prerequisites that allow organisms occupying higher trophic levels to occur in nutrient-poor glacial waters.

Aqueous eDNA can originate from both water and land-dwelling biota. The dominant invertebrate taxa recovered at the two proglacial sites, Odonata (dragonflies and damselflies) at PL01 and Chironomidae (non-biting midges) at PL05, both have aquatic (larvae) and terrestrial (adult) life cycle stages; therefore, the origin of the eDNA for those taxa could not be determined. The proglacial site PL01 was determined to be an unlikely habitat for carnivorous Odonata larvae because it had a particularly low water temperature and harbored few aquatic animals. Therefore, the Odonata eDNA at PL01 was most likely deposited by flying adult individuals. The lack of invertebrate signals at most proglacial sites also supports their unsuitability as invertebrate habitats. However, Chironomidae detected at the downstream site PL05 may represent stream-dwelling larvae, as morphology-based studies have often found various Chironomid larvae dominating benthic macroinvertebrate assemblages in glacial rivers across Europe.^14^^,^^47^^,^^48^

No vertebrates (fishes, birds, and mammals) were detected at proglacial sites, but they were detected at all lake sites, confirming that glacial headwaters, with their poor nutrient levels and low faunal abundance, are unfavorable habitats for large-bodied species. Overall, the sequential addition of different biological groups and increasingly complex community organization along glacier-fed waters reflect the environmental tolerances of different taxa and the ecological drivers of biodiversity that likely encompass both abiotic filtering and biotic interactions.^49^

### Environmental drivers and community assembly mechanisms

The assemblage composition of different biological groups showed distinct environmental correlation patterns across sites (Figure 6). Cyanobacteria, diatoms, and invertebrates responded to both spatial and water nutritional factors, but the specific patterns varied. The assemblage compositions of cyanobacteria, diatoms, and invertebrates were all correlated with longitude. As environmental variables were predominantly measured at lake sampling sites, and the overall water flow direction in Ranwu Lake is longitudinal, this correlation indicated that spatial distribution variations in those groups may be associated with habitat characteristics, such as hydrological and substrate conditions, at different localities within the lake. Cyanobacteria and invertebrate assemblages were additionally correlated with latitude, suggesting that their assemblages may be more susceptible to the influences of subtle habitat changes in comparison with diatom assemblages. Different groups also showed divergent correlations with water nutrients. While cyanobacteria and diatom compositions were related to a single nitrogen metric and a single carbon metric, respectively, invertebrate assemblages were found to be associated with four metrics of carbon, nitrogen, and phosphate. These findings suggest that the environmental responsiveness and habitat specificity of invertebrates may be greater than those of other groups.^50^^,^^51^^,^^52^ In contrast to the microbial and invertebrate communities, vertebrate assemblage composition showed no correlation with spatial or physiochemical water factors, likely because many of the detected vertebrate species were terrestrial animals with strong locomotion ability, which were less affected by spatial factors or water properties. Similar community response patterns among biological groups have been observed for other glacier-fed running waters, where water eDNA-detected microbial and invertebrate communities showed substantially stronger correlations with environmental (spatial, hydrological, and water physiochemical) variables in comparison with vertebrates.^12^

Disentangling the relative contributions of various ecological processes to community assembly is crucial for understanding drivers of biodiversity distribution patterns and forecasting future ecosystem trends in response to environmental changes.^53^^,^^54^ We found disparate mechanisms for cyanobacterial community assembly in different habitats (proglacier vs. Ranwu Lake) and for different biological groups in the same habitat (Figure 7). As the responses of different cyanobacteria taxa to environmental factors can vary greatly, and the proglacial and lake sites showed significant differences in cyanobacterial assemblage composition and environmental conditions, it is not surprising that the community assembly mechanisms for cyanobacteria differed between the two habitats. Cyanobacteria from Ranwu Lake showed primarily homogenous selection-driven assembly, perhaps due to the harsh, yet relatively uniform, environmental conditions in the lake, which may have led to strong and similar forms of selection pressure across the lake. Similarly, homogeneous selection has been shown to dominate microbial community assembly in other extreme and energy-restricted systems, such as glacier-fed streams,^55^ an oligotrophic gyre,^56^ and deep-water sediments.^57^ However, cyanobacterial assemblages at the proglacial sampling sites appeared to be an exception to this general pattern. The proglacial sites included a number of localities that showed high habitat heterogeneity, in which water hydrology and physiochemical properties changed rapidly as the distance from the glacier terminus increased. These changes may have led to considerable variations in the cyanobacterial assemblage compositions across the proglacial sites (see also Figure 4) and could have confounded the assembly mechanism analysis. Thus, the contributions of selection and dispersal were both low, while those of drift and other neutral processes dominated cyanobacterial assembly at proglacial sites.

In comparison with prokaryotic biota, the community assembly of eukaryotic biota has been less studied. Limited evidence has shown that, consistent with our result, ecological drift (neutral processes) primarily accounted for the community assembly of coastal phytoplankton^58^ and diatoms in a glacier-fed stream.^12^ Community assembly of diatoms in Ranwu Lake was mainly explained by drift, suggesting that the influences of environmental selection and dispersal on diatoms were limited. Of all biological groups assessed in our study, diatoms were the group with the highest number of OTUs detected. Different diatom species may have distinct environmental responses and dispersal ability, which lead to different distribution patterns within the lake. Therefore, the importance of deterministic processes (selection) was relatively low for diatoms in the cross-lake analysis, while community assembly appeared to be governed by stochastic processes (drift and dispersal). Few studies have examined invertebrate community assembly mechanisms in glacial habitats. Our analysis showed that invertebrates in the lake, similar to cyanobacteria from the same sites, had homogeneous selection-dominated assembly, which was indicative of a strong effect of environmental filtering on this group. Further investigations of other systems are needed to determine whether our findings represent a common pattern for invertebrates in high-altitude lakes. As the climate continues to warm and glaciers melt at a correspondingly faster rate, the relatively influences of different factors on the species composition and community assembly mechanisms of various biota inhabiting glacial environments will likely change.^59^ More in-depth study is necessary to fully understand the pattern and impact of these alterations.

### Knowledge gaps and future studies

Compared with those recovered from the lake sites, substantially larger proportions of OTUs detected in the proglacial samples could only be assigned to cyanobacteria, diatoms, and invertebrates at higher taxonomic levels, while they matched no existing reference sequences with high identity (Figure 2), indicating that the proglacial biodiversity on the Tibetan Plateau is considerably underrepresented in the available sequence databases. In addition to the high endemism characteristic of Tibetan Plateau biodiversity,^60^^,^^61^ the uniqueness of the glacier biota has been shown to be particularly high at the terminus zone.^10^ The extreme environmental conditions of the glacial terminus zone and the low habitat connectedness between glaciers likely give rise to specialized biota that are unique to each glacier, and challenges associated with assessing glacial fronts and obtaining biological samples further complicate the difficult task of constructing comprehensive glacial biodiversity databases. For instance, by sequencing metagenomes and cultured isolates from 21 Tibetan glaciers, Liu et al., 2022^62^ found that 88.3%–100% of 968 detected species-level microbial OTUs may represent novel species. Our finding that the proportion of unassigned sequences in proglacial samples was greater than that of lake samples substantiates the significant knowledge gap that currently exists regarding Tibetan glacial biodiversity.

The Tibetan Plateau has warmed 0.3°C/10 years over the past three decades, which is twice the global average warming rate over this period.^63^^,^^64^ Climate warming has considerably increased terrestrial primary production, accelerated deglaciation, and changed regional hydrological regimes on the Tibetan Plateau,^65^^,^^66^ all of which inevitably impact both terrestrial and aquatic biological communities and ecosystem processes. Glacier-fed waters and their associated biomes are sentinel systems that indicate early changes in the plateau ecosystem. For instance, diatom assemblage composition serves as a sensitive bioindicator of ecosystem integrity in glacial streams and lakes,^67^^,^^68^ which is also demonstrated in our IDP analysis (Figure 2). Further, warming can induce structural reforms in food webs through trophic cascades and other biotic interactions and ultimately impair glacial aquatic ecosystem functioning.^69^^,^^70^ Therefore, close monitoring of biological communities in glacial meltwater can be a sensitive tool to trace global change effects and gauge mitigation strategies.^69^^,^^71^

Our results shed light on the spatial succession of biological communities, from simple to complex, along glacial flowpaths and provide information regarding the environmental tolerances and requirements of different taxa. The highly heterogeneous community composition across glacier-fed water systems highlights the complexity and dynamic nature of glacier-influenced ecosystems, which remain largely uncharacterized for most glaciers on the Tibetan Plateau. A more extensive approach to profiling the plateau’s biodiversity is crucial for gaining a deeper understanding of its organization and dynamics at the community level. Moreover, further analyses linking multiple taxa to environmental alterations and climate warming across glacier-influenced habitats will provide essential information about the effects of global change on plateau biodiversity and aid prediction of future trends of glacial ecosystem processes, stability, and functions.

### Limitations of the study

Glacier-fed water systems are highly dynamic ecosystems that show significant seasonal fluctuations. Our study only investigated a single time point during the melt season, while more comprehensive sampling spanning annual variations in community composition will provide information on the temporal effect of glacial influence on the associated aquatic biota. Furthermore, the low species resolution of a large proportion of the organisms detected in the proglacial habitat hampers a thorough knowledge of the taxonomic identities and biological traits of the biota. More research efforts combining morphotaxonomy and DNA profiling would be necessary to fully understand the biodiversity in these understudied ecosystems.

## STAR★Methods

### Key resources table

**Table:** 

REAGENT or RESOURCE	SOURCE	IDENTIFIER
**Critical commercial assays**		

DNeasy Blood & Tissue Kit	Qiagen	Cat#69506
Premix Ex Taq™	TaKaRa	Cat#RR902A
MiniBEST DNA Fragment Purification Kit Ver.4.0	TaKaRa	Cat#9761
NEBNext® Ultra™ DNA Library Prep Kit for Illumina®	New England Biolabs	Cat#E7370L

**Deposited data**		

Raw Illumina sequencing data for multi-marker environmental DNA metabarcoding	This paper	NCBI SRA database BioProject PRJNA1045092, SRA data SRX22646161, SRX22646162, SRX22646163, and SRX22646164

**Oligonucleotides**		

Primers CYA for cyanobacteria	Monchamp et al., 2018^25^	Table S2
Primers 708F for diatoms	Chonova et al., 2019^26^	Table S2
Primers BF for invertebrates	Elbrecht and Leese, 2017^27^	Table S2
Primers Tele02 for vertebrates	Taberlet et al., 2018^28^	Table S2

**Software and algorithms**		

OBITools3	N/A	https://metabarcoding.org/obitools3
VSEARCH v.2.18.0	Rognes et al., 2016^72^	https://github.com/torognes/vsearch/releases
DADA2 v.1.24.0	Callahan et al., 2016^73^	https://benjjneb.github.io/dada2/
vegan v.2.6–2	Oksanen et al., 2022^74^	https://CRAN.R-project.org/package=vegan
DiaThor v.0.1.0.	Nicolosi Gelis et al., 2022^75^	https://CRAN.R-project.org/package=diathor
MEGA X	Kumar et al., 2018^76^	https://www.megasoftware.net/
IQ-TREE2	Minh et al., 2020^77^	http://www.iqtree.org/
iTOL v.5	Letunic and Bork, 2021^78^	https://itol.embl.de/
betapart v.1.5.6	Baselga and Orme, 2012^79^	https://CRAN.R-project.org/package=betapart
linkET v.0.0.5	Huang, 2021^80^	https://github.com/Hy4m/linkET
iCAMP v.1.5.12	Ning et al., 2020^81^	https://CRAN.R-project.org/package=iCAMP
R Statistical Software v.4.2.1	R Core Team, 2022^82^	https://www.r-project.org
ggplot2 v.3.4.1	Wickham, 2016^83^	https://CRAN.R-project.org/package = ggplot2

### Resource availability

#### Lead contact

Further information and requests for reagents should be directed to and will be fulfilled by the lead contact, Meng Yao (yaom@pku.edu.cn).

#### Materials availability

This study did not generate new unique reagents.

#### Data and code availability

• The sequencing data are available in NCBI Sequence Read Archieve (SRA) database, with the accession number listed in the key resources table.
• This paper does not report original code.
• Any additional information required to reanalyze the data reported in this paper is available from the lead contact upon request.

### Method details

#### Study area and sampling

Parlung (or Palong; PL) No. 4 glacier (PL; 29°14′ N, 96°55′ E; 4,650–4,964 m a.s.l.) and Ranwu Lake (29°27′N, 96°48′E; 3,920–3,930 m a.s.l.) are located in the upper Parlung-Zangbo River Basin in the southeastern Tibetan Plateau. The study region is characterized by a plateau with a cold temperate climate and mean annual precipitation of about 552 mm. The PL is a typical mountain valley glacier that flows north-eastward, has an area of 11.7 km^2^ and a length of 8 km,^84^ and is affected by the Indian summer monsoon. Due to an ongoing warming trend (mean of 0.39°C/decade since 1990), its mass loss rate has been accelerating.^24^ Ranwu Lake, a glacier-fed freshwater lake, is 29 km long, about 0.8 km wide on average, and has a 1,985 km^2^ total catchment area, of which 16.8% is occupied by glaciers.^85^ From the end of December to the middle of March, the lake is completely ice-covered. The Qurihe River, fed mainly by meltwater from PL, is one of the lake’s main inflowing rivers, and the lake’s single outflow is a headwater of the Parlung-Zangbo River.^86^

We conducted systematic water sampling in summer 2019 over a two-day period (July 16 and 17). The sampling was conducted at the proglacial lake at PL’s terminus (PL01) and its outflow (PL02; geographic distance 0.37 km to PL01), and along the adjacent meltwater stream (PL03, PL04, and PL05; 0.81, 1.24 and 1.76 km to PL01, respectively). These five sites are collectively referred to as the ‘proglacial sites’. Additionally, we sampled water at 11 sites (RW01–RW11) at Ranwu Lake (referred to as the ‘lake sites’; Figure 1). The site code numbers increased with the direction of flow. Along with water sample collection, we recorded the latitude, longitude, and elevation of each site using a handheld GPS device. We measured 10 physiochemical parameters at six representative sites (PL01, RW03, RW06, RW08, RW10, and RW11): water temperature (Tem), pH, dissolved oxygen (DO), dissolved organic carbon (DOC), dissolved organic nitrogen (DON), ammonium nitrogen (NH_4_^+^-N), nitrate nitrogen (NO_3_^−^-N), total carbon (TC), total nitrogen (TN), and total phosphorus (TP) (Table S1). On-site pH measurements were made with a pocket pH meter (Hanna Instruments, Woonsocket, Rhode Island, USA), while Tem and DO were measured using a JPB-607 portable DO meter (INESA Scientific Instrument Co. Ltd, China). The remaining parameters were measured in the laboratory according to standard methods, as previously described in Zhang et al., 2021^87^ The weather conditions on the sampling days were similar, with overcast skies and without any significant precipitation or hydrological events that could have affected the results.

We collected 1 L of surface water at each site in a sterile plastic bottle. Measurements of the geographical information and water physiochemical parameters at six representative sites (shown in Table S1) are described in the STAR methods. All water samples were stored in a cold box and each was filtered within a few hours through a 0.45-μm Sterivex filter unit (Millipore Corporation, Billerica, Massachusetts, USA) using a peristaltic pump and sterile tubing. New tubing was used for each sample. To monitor contamination, filtration blanks using distilled water were processed alongside the water samples. Following filtration, 1 mL of 95% ethanol was injected into each filter cartridge. Each filter cartridge was capped, individually packaged in a clean ziplock bag, and transported to the laboratory at Peking University for further processing.

#### Environmental DNA laboratory procedures

All extractions were conducted in a dedicated eDNA extraction room. Prior to each extraction, laboratory tools and surfaces were pre-treated with a 10% bleach solution, followed by an application of DNA Away (Molecular BioProducts, San Diego, California, USA). For DNA extraction from the Sterivex filters, the cartridges were carefully opened using a sterilized PVC pipe cutter, and each filter was removed from the cartridge and dried under aseptic conditions. Each filter was subsequently cut into smaller pieces (2–3 mm^2^) and transferred into a sterile 2-mL microcentrifuge tube. Total DNA was extracted from each filter using the DNeasy Blood and Tissue Kit (Qiagen GmbH, Hilden, Germany) following a modified protocol.^88^^,^^89^ Briefly, 720 μL of ATL buffer and 50 μL of Proteinase K were added to the tube. The filter pieces were incubated at 56°C for 2.5 h. Subsequent steps were conducted following the extraction kit’s recommendations. The final step involved eluting the extracted DNA using 100 μL of AE buffer. Extraction blanks containing no filters were conducted in parallel with each batch of extractions.

Four metabarcoding primer sets were used to amplify the eDNA extracts, with each targeting a specific biological group (Table S2). For each eDNA extract, PCR was performed in triplicate. PCR amplifications were carried out in a 25 μL reaction mixture, which consisted of 2.5 μL eDNA extract (diluted 5-fold to reduce PCR inhibition), 0.5 μL of 10 μM forward and reverse tagged primers, 0.5 μL of 20 mg/mL bovine serum albumin, 12.5 μL of 2× Premix Ex Taq (Takara Bio Inc., Kusatsu, Japan), and 8.5 μL of double-distilled water. To facilitate identification of the individual PCR products, unique eight variable nucleotides (e.g., tags) were added to the 5′ ends of both the forward and reverse primers of each PCR.^90^ The cycling conditions for PCR mostly followed the original publications of the primers (Table S2). For CYA primers, the PCR program included an initial denaturation at 95°C for 10 min, followed by 30 cycles at 95°C for 15 s, 60°C for 30 s, and 72°C for 45 s, with a final extension at 72°C for 5 min. For 708F primers, the cycling conditions comprised an initial denaturation at 95°C for 15 min, followed by 35 cycles at 95°C for 45 s, 55°C for 45 s, and 72°C for 45 s. For BF primers, the cycling conditions included an initial denaturation at 95°C for 5 min, followed by 40 cycles at 95°C for 30 s, 50°C for 45 s, and 68°C for 45 s, with a final extension at 72°C for 10 min. Lastly, for Tele02 primers, the PCR program involved an initial denaturation at 95°C for 10 min, followed by 45 cycles at 95°C for 30 s, 63°C for 45 s, and 72°C for 60 s, with a final extension at 72°C for 10 min. Negative controls consisting of filtration blanks, extraction blanks, and no-template PCR blanks were included in each PCR run to monitor for potential contamination at different stages.

PCR products were initially analyzed using 1.5% agarose gel electrophoresis to confirm successful amplification. Subsequently, the PCR products of the same primers were pooled in equal volumes (20 μL each) and purified using the MiniBEST DNA Fragment Purification Kit (Takara Bio, Shiga, Japan). A separate sequencing library was constructed with the PCR products of each primer set. Library construction was performed by the Novogene company (Novogene, Beijing, China) using a PCR-free protocol with the NEBNext Ultra DNA Library Prep Kit for Illumina (New England Biolabs, Ipswich, Massachusetts, USA). Paired-end sequencing (2 × 250 bp for the CYA, 708F, and BF libraries; 2 × 150 bp for the Tele02 library) was performed on the Illumina NovaSeq 6000 platform (Illumina Inc., San Diego, California, USA).

#### Bioinformatics processing and taxonomic assignment

Each sequencing library underwent distinct bioinformatics processing using the OBITools3 package (https://metabarcoding.org/obitools3)^91^ to process the raw Illumina-sequencing reads following a series of sequence filtering steps. Specifically, *alignpairedend* was employed to align and assemble the forward and reverse sequencing reads, after which sequences with low overlap alignment scores (≤0.6) were eliminated using *grep*. Demultiplexing was achieved using *ngsfilter* to assign each sequence to its corresponding sample, while *obiuniq* was used to dereplicate identical sequences. Only sequences with a total count ≥10 and a length longer or equal to the minimum length (CYA: 350 bp; 708F: 250 bp; BF: 300 bp; Tele02: 120 bp) were retained using *obigrep*. Finally, *obiclean* was applied to detect and eliminate sequences that were potentially generated due to sequencing errors or PCR amplification artifacts (r = 0.5). Putative chimeric sequences were eliminated using the *uchime3_denovo* command in VSEARCH.^72^ The remaining sequences were then clustered into OTUs at a 97% similarity threshold through the application of *sumaclust* (http://metabarcoding.org/sumaclust).

We used the *assignTaxonomy* function in the R package DADA2^73^ to assign the cyanobacteria and diatom taxonomies. The SILVA (release 138) reference database was used to classify cyanobacterial sequences, and only OTUs belonging to the phylum Cyanophyta were retained. Both chloroplast OTUs and cyanobacterial OTUs lacking taxonomic information at the class level were also removed. The Diat.barcode (v.10) database^92^ was used for diatom taxonomic assignments. The diatom OTUs were initially filtered to include only those belonging to the phylum Bacillariophyta, and only those OTUs with class-level taxonomic information were subsequently retained.

Sequence alignment for invertebrates was conducted using MIDORI ref. 2 (http://www.reference-midori.info/index.html) against the GenBank eukaryotic mitochondrial COI database (release 251).^93^ All OTUs belonging to the metazoan kingdom (Kingdom Animalia) were initially retained, and then those classified under the subphylum Vertebrata or lacking phylum information were excluded. To enhance identification accuracy, the remaining OTUs were re-matched to the BOLD database (http://www.boldsystems.org/) using the JAMP method (https://github.com/VascoElbrecht/JAMP) with the Boldigger package,^94^ as described previously.^95^ The subsequent results were combined with the Naive Bayesian classifier results to obtain more detailed taxonomic information.

For vertebrate sequence alignment, we utilized MIDORI ref. 2 against the GenBank eukaryotic mitochondrial 12S (srRNA) database. Only OTUs belonging to the Vertebrata subphylum and possessing taxonomic information at the order level were retained for further analysis, and OTUs identified as human sequences were removed from the dataset. To enhance identification accuracy, the remaining OTUs were re-matched to the NCBI nonredundant sequence database using the BLASTn program (https://blast.ncbi.nlm.nih.gov/Blast.cgi). If a query sequence matched a single locally occurring species in the database with ≥98% identity and 100% coverage, that species was assigned to the corresponding OTU. In cases where a query matched multiple local species with ≥98% identity and 100% coverage, the lowest taxonomic level encompassing all matched species was assigned to the OTU. In addition, OTUs with the same taxonomic name were collapsed. Local species records were accrued from past survey records for fishes,^96^ birds,^97^ and mammals,^98^ as well as available records from the Global Biodiversity Information Facility (https://www.gbif.org/). The sequences of the domesticated pig and dog could not be differentiated from those of their respective wild ancestors, the wild boar (*Sus scrofa*) and gray wolf (*Canis lupus*). As wild boars and wolves occur in the study area, we tentatively assigned the sequences to these wild species rather than their domesticated counterparts.

After the taxonomic assignments, the highest count of each OTU in the negative control PCRs of a given library was subtracted from the eDNA PCR results to remove potential contamination. Furthermore, to mitigate the potential impact of tag jumping, any OTU with a read count <10 in each PCR product was eliminated.^99^

### Quantification and statistical analysis

To assess the impact of sequencing depth on the observed OTU richness within each group, we used the rarecurve function in the R package vegan v.2.6–2 to construct rarefaction curves.^74^ PCR results that had low sequence counts in each group (CYA: <300; 708F: <5,000; BF: <50; Tele02: <5,000) were excluded from subsequent analysis. The predetermined thresholds for inclusion were based on the rarefaction curves (Figure S1) and were consistent with a previous report.^12^ After all sequence filtering was complete, 47 PCR results were retained for cyanobacteria, 39 for diatoms, 33 for invertebrates, and 28 for vertebrates. Next, the OTU data were normalized using the total sum normalization method^100^ to calculate each PCR’s RRA, which is the number of reads for a specific OTU divided by the total number of reads of the PCR, averaged across the PCR replicates of each sample.

Diatom-based biological indices are widely employed to assess water quality changes in freshwater environments. The IDP is an effective diatom-based indicator of organic pollution and eutrophication, and it ranges from 0 (low levels of nutrients and organic enrichment) to 4 (high concentrations of organic matter).^29^ We calculated the IDP using the R package DiaThor v.0.1.0.^75^

Compositional variation between sites (i.e., β diversity) for different biological groups was quantified using the Bray–Curtis dissimilarity index, which was estimated using the RRA data with the *vegdist* function in vegan. To assess community compositional differences, we conducted PERMANOVA using the *adonis2* function in vegan with 9,999 permutations. The results were visualized using the vegan *metaMDS* function, which employs NMDS analysis based on Bray–Curtis dissimilarity.

To estimate the phylogenetic relationships among the OTUs of each of the four biological groups, we first conducted multiple sequence alignment of representative OTU sequences using the *Muscle* method in MEGA X.^76^ Subsequently, IQ-TREE2^77^ was used to infer the maximum-likelihood tree, and the best substitution model was selected using the “Auto” parameter. The tree was visualized and annotated using the Interactive Tree Of Life webtool.^78^ To gain insights into the specific OTUs that contributed the most to the observed dissimilarities between the proglacial and lake communities within each biological group, we used the *simper* function in vegan to conduct a SIMPER analysis.

The β-diversity for different biological groups was partitioned into species turnover and nestedness components using the *beta.multi* function for Sørensen (qualitative) dissimilarity and the *beta.multi.abund* function for Bray–Curtis (quantitative) dissimilarity in the R package betapart v.1.5.6.^79^

To identify factors that may influence biological communities, Mantel tests were employed to assess the correlations between biotic assemblage compositional similarity and environmental factors across different sites. The environmental factors encompassed three spatial variables (latitude, longitude, and elevation) and 10 physiochemical water parameters (Table S1). The spatial and environmental data were logarithmically transformed to mitigate skewness, and the RRA data underwent Hellinger transformation prior to analysis. A heatmap was generated in the R package linkET v.0.0.5^97^ to visualize the correlations between assemblage compositions and environmental factors.

Both deterministic (i.e., niche-based, such as environmental filtering and biological interactions) and stochastic (including birth/death, dispersal, speciation, and ecological drift) processes can influence community diversity and dynamics, and determining the relative importance of these processes is a central theme in community ecology.^53^^,^^101^^,^^102^ To investigate the community assembly mechanisms of different biological groups, a phylogenetic-bin-based null model was applied with the R package iCAMP v.1.5.12.^81^ By using iCAMP, the relative importance of five assembly processes (homogeneous selection, heterogeneous selection, dispersal limitation, homogenizing dispersal, and drift and others) was quantified based on all pairwise comparisons between samples.^103^ Phylogenetic compositional variation among assemblages is expected to be low under homogeneous selection (i.e., selection under similar abiotic and biotic conditions), whereas heterogeneous selection leads to a high level of phylogenetic compositional variation. Similarly, homogenizing dispersal refers to the situation where a high dispersal rate leads to low taxonomic compositional variation among assemblages, while dispersal limitation (low dispersal rate) increases community taxonomic variation. Processes other than strong selection and dispersal are collectively designated as drift.^81^ Therefore, by analyzing the taxonomic (OTU) and phylogenetic (sequence) compositional variations among samples, the relative contributions of different assembly processes can be assessed. The iCAMP analysis was performed using the RRA data and sequences of the detected OTUs and the *icamp*.*big* function with a general threshold of 0.2 for phylogenetic signal distance, a randomization number of 1,000, and a minimal bin size determined by the *ps*.*bin* function. We conducted this analysis for proglacial and lake samples separately, as these two habitats each showed distinct community compositions for each biological group (see results). Because only cyanobacteria were detected at all proglacial sites, while the other groups were detected at only 0–3 proglacial sites, we assessed proglacial habitat community assembly only for cyanobacteria. Vertebrates were not considered in this analysis because their community assemblies may have been subjected to other variables unfit for iCAMP assumptions.

All statistical analyses were performed in the R v.4.2.1 environment.^82^ Statistically significant differences were determined at p values < 0.05. Figure 1 was created with the open source software QGIS v.3.22 (https://www.qgis.org). Figure 2 was drawn using the open source software PAST v.4.13 (https://www.nhm.uio.no/english/research/resources/past/). Figures 3, 4, and 7, S1, and S2 were generated using the R package ggplot2 v.3.4.1.^83^ Figure 5 was generated using the online tool Interactive Tree of Life v5 (available at https://itol.embl.de). Figure 6 was generated using the R package linkET v.0.0.5 (https://github.com/Hy4m/linkET). All final figure layouts were then adjusted and improved with Adobe Illustrator v.22.1 (https://www.adobe.com).

## Supplemental information

Document S1. Figures S1 and S2 and Tables S1–S6

Data S1. Summary of the detected operational taxonomic units (OTUs)
