# Supplementary material for: Successive accumulation of biotic assemblages at a fine spatial scale along glacier-fed waters
Source: iScience. 2024 Mar 26;27(4):109476. doi: 10.1016/j.isci.2024.109476 (PMC11015461; doi:10.1016/j.isci.2024.109476)
Supplement: Document S1. Figures S1 and S2 and Tables S1–S6 [file mmc1.pdf]

iScience, Volume *27*

Supplemental information

Successive accumulation of biotic assemblages at a fine spatial scale along glacier-fed waters

Qi Lu, Yongqin Liu, Jindong Zhao, and Meng Yao

Supplemental Tables

Table S1 Location and water physiochemical parameters for six representative sites, Related to Figures 1 and 6.

Sampling site	Longitude (E)	Latitude (N)	Elevation (m)	DO (mg/L)	Tem (°C)	pH	DOC (mg/L)	DON (mg/L)	NH_4^+-N (mg/L)	NO_3^--N (mg/L)	TC (mg/L)	TN (mg/L)	TP (mg/L)
PL01	96.9354	29.2657	4647	5.15	7.58	7.85	0.51	0.10	0.06	0.18	12.51	0.28	0.05
PL02	96.9384	29.2678	4632	—	—	—	—	—	—	—	—	—	—
PL03	96.9418	29.2704	4630	—	—	—	—	—	—	—	—	—	—
PL04	96.9429	29.2747	4604	—	—	—	—	—	—	—	—	—	—
PL05	96.9454	29.2789	4585	—	—	—	—	—	—	—	—	—	—
RW01	96.8351	29.3853	3915	—	—	—	—	—	—	—	—	—	—
RW02	96.8394	29.3959	3922	—	—	—	—	—	—	—	—	—	—
RW03	96.8384	29.4003	3921	5.37	9.12	7.62	0.46	0.09	0.05	0.35	19.38	0.44	0.03
RW04	96.8386	29.4049	3930	—	—	—	—	—	—	—	—	—	—
RW05	96.8020	29.4424	3925	—	—	—	—	—	—	—	—	—	—
RW06	96.7835	29.4786	3922	5.40	8.57	7.67	0.33	0.07	0.01	0.23	19.36	0.30	0.02
RW07	96.7724	29.4973	3919	—	—	—	—	—	—	—	—	—	—
RW08	96.7353	29.5050	3931	5.15	11.30	7.65	0.29	0.06	0.01	0.31	12.20	0.38	0.01
RW09	96.7103	29.4925	3925	—	—	—	—	—	—	—	—	—	—
RW10	96.6732	29.4894	3925	5.32	9.70	7.40	0.34	0.07	0.01	0.25	10.13	0.31	0.02
RW11	96.6540	29.4819	3919	5.66	9.63	7.67	0.32	0.07	0.01	0.24	11.14	0.31	0.02

DO, dissolved oxygen; Tem, water temperature; DOC, dissolved organic carbon; DON, dissolved organic nitrogen; NH_4^+-N, ammonium nitrogen; NO_3^--N, nitrate nitrogen; TC, total carbon; TN, total nitrogen; TP, total phosphorus.

Table S2 Primer information for eDNA metabarcoding, Related to Figure 2.

Target group	Primer name	Gene region	Primer sequence (5′–3′)	Target size (bp)	Reference
**Cyanobacteria**	CYA	16S	F: GGGGAATYTTCCGCAATGGG<br>R: ACTACWGGGGTATCTAATCCC	386	Monchamp et al., 2018[1]
**Diatoms**	708F	rbcL	F (708F): AGGTGAAGTTAAAGGTTCATACTTDAA<br>R (R3): CCTTCTAATTTACCAACAACTG	263	Chonova et al., 2019[2]
**Invertebrates**	BF	COI	F (BF1): ACWGGWTGRACWGTNTAYCC<br>R (BR2): TCDGGRTGNCCRAARAAYCA	316	Elbrecht and Leese, 2017[3]
**Vertebrates**	Tele02	12S	F: AAACTCGTGCCAGCCACC<br>R: GGGTATCTAATCCCAGTTTG	167	Taberlet et al., 2018[4]

F, forward primer; R, reverse primer.

Table S3 The numbers and percentages of the detected operational taxonomic units (OTUs) assigned at various taxonomic levels for each biological group, Related to Figure 2.

Group	Total OTUs	Phylum	Class	Order	Family	Genus	Species
Cyanobacteria (phylum Cyanophyta)	125	—	24 (19.2%)	11 (8.8%)	22 (17.6%)	68 (54.4%)	—
Diatoms (phylum Bacillariophyta)	316	—	51 (16.1%)	20 (6.3%)	36 (11.4%)	81 (25.6%)	128 (40.5%)
Invertebrates (Metazoa except Vertebrata)	183	18 (9.8%)	30 (16.4%)	24 (13.1%)	37 (20.2%)	43 (23.5%)	31 (16.9%)
Vertebrates (subphylum Vertebrata)	18	—	—	—	1 (5.5%)	3 (16.7%)	14 (77.8%)

Table S4 Taxonomic coverage of the detected OTUs (number of different categories to which they belong) at each taxonomic level, Related to Figure 2.

Group	Phylum	Class	Order	Family	Genus	Species
Cyanobacteria	1	3	12	16	38	—
Diatoms	1	3	10	20	42	73
Invertebrates	6	14	21	34	48	28
Vertebrates	1	3	8	11	15	14

Table S5 Number of OTUs detected at individual sampling sites, Related to Figure 2.

Group	PL 01	PL 02	PL 03	PL 04	PL 05	RW 01	RW 02	RW 03	RW 04	RW 05	RW 06	RW 07	RW 08	RW 09
Cyanobacteria	13	34	12	31	16	26	10	32	36	57	34	48	40	38
Diatoms	0	0	4	3	26	110	131	85	130	136	45	111	73	42
Invertebrates	6	0	0	0	3	41	24	17	2	18	13	62	28	40
Vertebrates	0	0	0	0	0	8	8	4	7	7	3	7	5	4

Table S6 Results of the similarity percentage analysis, Related to Figure 5.

OTU	Average dissimilarity	Contribution (%)	Cumulative (%)	Mean RRA proglacier	Mean RRA lake
**Cyanobacteria**					
Cyanobium_PCC-6307	39.5	43.8	43.8	0.0%	79.0%
Cyanobacteriia_OTU3	10.4	11.5	55.3	22.1%	2.3%
Leptolyngbyaceae_OTU6	8.4	9.3	64.6	18.2%	2.6%
Cyanobacteriia_OTU5	7.9	8.8	73.4	17.5%	1.9%
Pseudanabaena_PCC-7429	4.2	4.7	78.0	6.8%	4.9%
Leptolyngbyaceae_OTU4	2.6	2.8	80.9	3.6%	4.3%
Sericytochromatia_OTU21	2.4	2.7	83.5	4.8%	0.0%
Aliterella_OTU15	1.9	2.1	85.6	3.8%	0.1%
Chamaesiphon_PCC-7430	1.8	2.0	87.7	3.4%	1.4%
Pseudanabaena_PCC-7429_OTU11	1.6	1.8	89.4	3.2%	0.1%
Synechococcus_PCC-7502	1.2	1.4	90.8	2.4%	0.1%
Pseudanabaenaceae_OTU22	1.2	1.3	92.1	2.3%	0.2%
Sericytochromatia_OTU34	1.0	1.1	93.1	2.0%	0.0%
**Diatoms**					
Cymbellales_OTU11	20.8	27.7	27.7	43.5%	2.0%
Fragilaria_acus/radians_complex	14.7	19.6	47.4	25.8%	41.2%
Encyonema_minutum	11.2	14.9	62.3	23.2%	2.6%
Stephanodiscus_hantzschii	10.5	14.1	76.4	0.0%	21.1%
Cymbella_neocistula	4.1	5.5	81.9	1.3%	6.7%
Hannaea_arcus	3.1	4.1	86.0	5.6%	5.4%
Ulnaria_ulna	1.6	2.2	88.2	0.0%	3.3%
Diatoma_moniliformis	1.6	2.2	90.4	0.1%	3.3%
Nitzschia_dissipata_var._media	1.4	1.8	92.2	0.0%	2.7%
Achnanthidium_minutissimum	0.8	1.0	93.2	0.2%	1.7%
**Invertebrates**					
Lepidoptera_OTU2	12.6	12.6	12.6	0.0%	25.1%
Insecta_OTU47	12.5	12.5	25.1	25.0%	0.0%
Chironomidae_OTU8	10.0	10.0	35.0	19.9%	0.0%
Insecta_OTU88	8.0	8.0	43.0	15.9%	0.0%
Insecta_OTU4	6.9	6.9	49.8	0.0%	13.7%
Insecta_OTU57	6.2	6.2	56.0	12.3%	0.0%
Lepidoptera_OTU3	4.9	4.9	60.9	0.0%	9.8%
Odonata_OTU119	4.5	4.5	65.4	9.1%	0.0%
Insecta_OTU1	4.1	4.1	69.5	0.0%	8.2%
Arthropoda_OTU83	3.8	3.8	73.3	7.5%	0.0%
Insecta_OTU11	2.5	2.5	75.8	5.1%	0.0%
Metacnephia_OTU12	2.0	2.0	77.8	0.0%	4.0%
Insecta_OTU9	1.7	1.7	79.6	0.0%	3.5%
Trombidiformes_OTU111	1.6	1.6	81.1	3.1%	0.0%
Lepidoptera_OTU18	1.1	1.1	82.2	0.0%	2.2%
Arthropoda_OTU146	1.0	1.0	83.3	2.1%	0.0%

Supplemental Figures

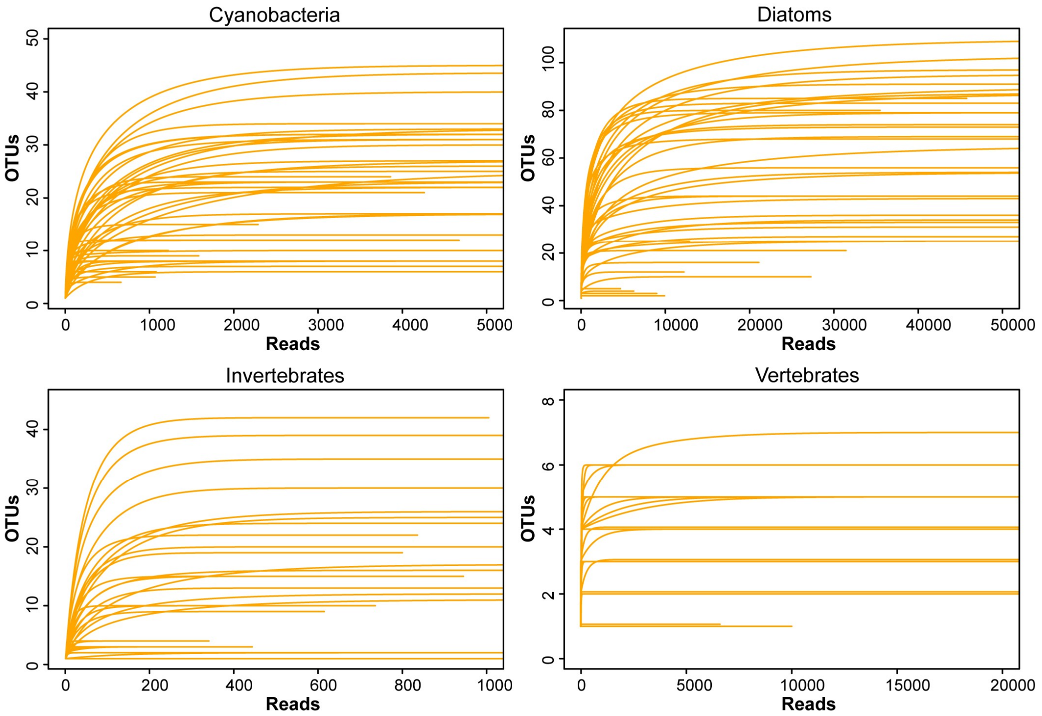

Figure S1 Rarefaction curves for the PCR results for four biological groups. The mean reads per PCR for each community were as follows: cyanobacteria, 15,687; diatoms, 114,940; invertebrates, 1,067; and vertebrates, 60,740, Related to Figure 2.

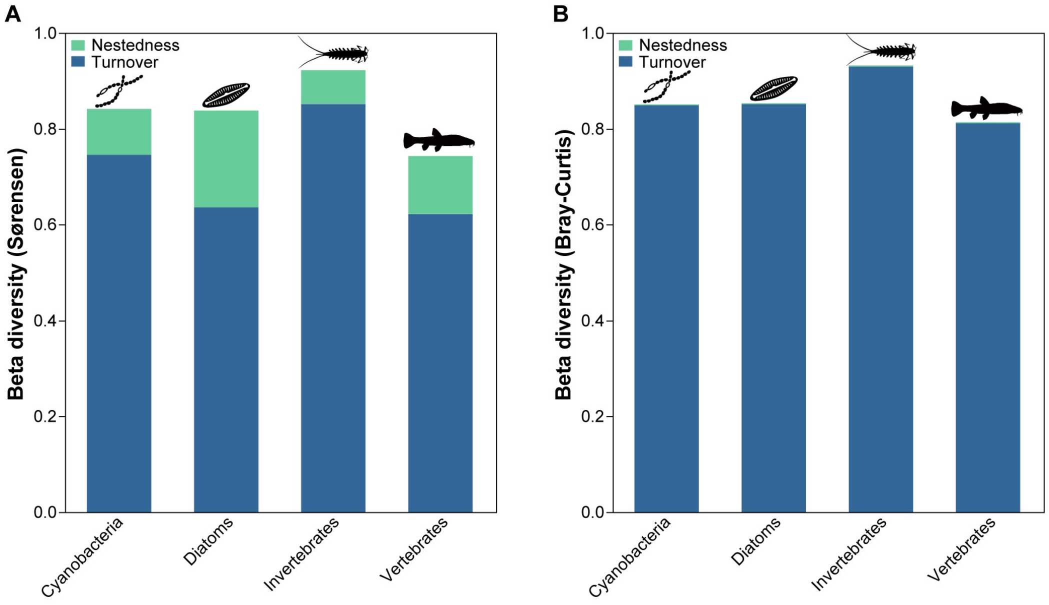

Figure S2 Relative contributions of turnover and nestedness component to total β diversity across all sites based on (A) Sørensen (qualitative) and (B) Bray–Curtis (quantitative) dissimilarity, Related to Figure 6.

Supplemental References

1. Monchamp, M.-E., Spaak, P., Domaizon, I., Dubois, N., Bouffard, D., and Pomati, F. (2018). Homogenization of lake cyanobacterial communities over a century of climate change and eutrophication. Nat. Ecol. Evol. *2*, 317-324. https://doi.org/10.1038/s41559-017-0407-0.

2. Chonova, T., Kurmayer, R., Rimet, F., Labanowski, J., Vasselon, V., Keck, F., Illmer, P., and Bouchez, A. (2019). Benthic diatom communities in an Alpine river impacted by waste water treatment effluents as revealed using DNA metabarcoding. Front. Microbiol. *10*, 653. https://doi.org/10.3389/fmicb.2019.00653.

3. Elbrecht, V., and Leese, F. (2017). Validation and development of COI metabarcoding primers for freshwater macroinvertebrate bioassessment. Front. Environ. Sci. *5*, 11. https://doi.org/10.3389/fenvs.2017.00011.

4. Taberlet, P., Bonin, A., Zinger, L., and Coissac, E. (2018). Environmental DNA: For Biodiversity Research and Monitoring (Oxford University Press).
